# Assessment of Placebo Response in Objective and Subjective Outcome Measures in Rheumatoid Arthritis Clinical Trials

**DOI:** 10.1001/jamanetworkopen.2020.13196

**Published:** 2020-09-16

**Authors:** Jan Vollert, Nancy R. Cook, Ted J. Kaptchuk, Shiv T. Sehra, Deirdre K. Tobias, Kathryn T. Hall

**Affiliations:** 1 Brigham and Women’s Hospital, Harvard Medical School, Boston, Massachusetts; 2 Pain Research, Department of Surgery and Cancer, Faculty of Medicine, Imperial College London, London, United Kingdom; 3 Beth Israel Deaconess Medical Center, Harvard Medical School, Boston, Massachusetts; 4 Mount Auburn Hospital, Harvard Medical School, Cambridge, Massachusetts

## Abstract

**Importance:**

Large placebo responses in randomized clinical trials may keep effective medication from reaching the market. Primary outcome measures of clinical trials have shifted from patient-reported to objective outcomes, partly because response to randomized placebo treatment is thought to be greater in subjective compared with objective outcomes. However, a direct comparison of placebo response in subjective and objective outcomes in the same patient population is missing.

**Objective:**

To assess whether subjective patient-reported (pain severity) and objective inflammation (C-reactive protein [CRP] level and erythrocyte sedimentation rate [ESR]) outcomes differ in placebo response.

**Design, Setting, and Participants:**

The placebo arms of 5 double-blind, randomized, placebo-controlled clinical trials were included in this cross-sectional study. These trials were conducted internationally for 24 weeks or longer between 2005 and 2009. All patients with rheumatoid arthritis randomized to placebo (N = 788) were included. Analysis of data from these trials was conducted from March 27 to December 31, 2019.

**Intervention:**

Placebo injection.

**Main Outcomes and Measures:**

The difference (with 95% CIs) from baseline at week 12 and week 24 on a 0- to 100-mm visual analog scale to evaluate the severity of pain, CRP level, and ESR.

**Results:**

Of the 788 patients included in the analysis, 644 were women (82%); mean (SD) age was 51 (13) years. There was a statistically significant decrease in patient-reported pain intensity (week 12: −14 mm; 95% CI, −12 to −16 mm and week 24: −20 mm; 95% CI, −16 to −22 mm). Similarly, significant decreases were noted in the CRP level (week 12: −0.51 mg/dL; 95% CI, −0.47 to −0.56 mg/dL and week 24: −1.16 mg/dL; 95% CI, −1.03 to −1.30 mg/dL) and ESR (week 12: −11 mm/h; 95% CI, −10 to 12 mm/h and week 24: −25 mm/h; 95% CI, −12 to −26 mm/h) (all *P* < .001).

**Conclusions and Relevance:**

The findings of this study suggest that improvements in clinical outcomes among participants randomized to placebo were not limited to subjective outcomes. Even if these findings could largely demonstrate a regression to the mean, they should be considered for future trial design, as unexpected favorable placebo responses may result in a well-designed trial becoming underpowered to detect the treatment difference needed in clinical drug development.

## Key Points

**Question:**

Are subjective patient-reported outcomes vs objective biomarkers associated with higher placebo responses in clinical trials?

**Findings:**

In this cross-sectional study examining the placebo arms of 5 randomized clinical trials of rheumatoid arthritis including 788 patients, objective markers of inflammation and subjective pain ratings improved in a comparable clinically meaningful magnitude. Baseline values were associated with placebo response, suggesting that regression to the mean might dominate response to randomized placebo treatment.

**Meaning:**

The findings of this study suggest that investigators may need to improve their understanding of natural history and baseline levels of outcomes because these factors can be important contributors to the response in placebo arms.

## Teaser

This cross-sectional study examines subjective vs objective responses to placebo administration in clinical trials on patients with rheumatoid arthritis.

## Introduction

Variability in the magnitude of clinical responses in the placebo arms of randomized clinical trials (RCTs) can result in effective drugs failing to reach the clinic. Placebo arms in RCTs are designed to control for the nonspecific effects of enrollment, including standard of care, regression to the mean, natural history of the disease, and placebo effects.^1^ Placebo arms should be kept as small as possible to avoid withholding potentially effective treatment from patients.^2^ It is important to distinguish between the placebo effect (a distinct physiological response to the therapeutic context and treatment delivery)^3^ and the placebo response in a clinical trial (improvement of patients in a placebo arm for any reason). Placebo effects are particularly strong in subjective outcomes (eg, pain),^4^ functional conditions (eg, irritable bowel syndrome),^5^ and fatigue,^6^ leading to the idea that placebo responses in clinical trials could be reduced by using objective biomarkers as primary outcomes rather than patient-reported outcomes. This approach, however, is indirect, since little is known about how objective and subjective outcomes vary in the placebo arms of RCTs^7^ and the approach ignores the role of other factors in the generation of the placebo response observed in an RCT. To directly compare placebo responses in objective biomarkers and subjective patient-reported outcomes, rheumatoid arthritis RCTs are ideal, as they usually collect data for assessment of patient-reported pain levels as well as changes in objective biomarkers (C-reactive protein [CRP] levels or erythrocyte sedimentation rate [ESR]),^8^ enabling insights into the magnitude of placebo effects within the placebo response in a homogeneous patient population. In the present study, we analyzed outcomes in the placebo arms of 5 RCTs of rheumatoid arthritis, comparing CRP level, ESR, and subjective pain levels. We hypothesized that, if the placebo effect is a leading contributor to the placebo response in these trials, the placebo response will be higher in pain levels, which are considered to be subjective compared with objective biomarkers. If, however, the placebo effect is superimposed by natural history disease fluctuation, regression to the mean phenomena, and other contributors to the placebo response in clinical trials, one would expect to note only minor differences between objective and subjective outcomes.

## Methods

All included studies were separately approved by the respective ethical boards. Per the Common Rule, this study was exempt from institutional review board review owing to use of database information. We extracted individual patient- and study-level data from the placebo and standard of care arm of available RCTs of rheumatoid arthritis providing pain, CRP, and ESR measures in the TransCelerate Biopharma placebo and standard of care database as of October 30, 2018. At the time it was shared, this database included contributions of anonymized or pseudonymized data from Allergan, Amgen, AstraZeneca, Eli Lilly, GlaxoSmithKline, Johnson & Johnson, Pfizer, Roche, and UCB Pharma. Contributions to the database were performed on a voluntary basis and did not follow comprehensive inclusion criteria. We included all studies in the database performed involving patients with rheumatoid arthritis of at least 24 weeks’ duration, providing individual patient-level data on at least (1) pain levels at baseline, week 12, and week 24; (2) CRP levels at baseline, week 12, and week 24; and (3) ESR at baseline, week 12, and week 24. Five studies fulfilled these inclusion criteria,^9,10,11,12,13^ all conducted between 2005 and 2009, with results being reported between 2007 and 2015. Analysis of data from these trials was conducted from March 27 to December 31, 2019. Study characteristics and size of the patient cohorts can be found in Table 1. Using the study identifiers extracted from the TransCelerate database to identify ClinicalTrials.gov records, information on general study design, study location, and treatment vehicle were extracted from ClinicalTrials.gov and study publications (Table 1, publicly available data). Because these data are proprietary, the data of this analysis are not shared publicly, and no public involvement in this study was possible. This study followed the Strengthening the Reporting of Observational Studies in Epidemiology (STROBE) reporting guideline for cross-sectional studies as applicable.

**Table 1.** Placebo Arms From the 5 Rheumatoid Arthritis Trials Included in This Study

Variable	Value	Trial				
		1	2	3	4	5
**Data set of this analysis**						
Total participants, including treatment arms, No.	2996	950	590	194	512	750
Participants randomized to placebo in this analysis, No.	788	199	101	88	156	244
Women, No. (%)	644 (82)	167 (84)	84 (83)	69 (78)	134 (86)	190 (78)
Age at baseline, mean (SD), y	51 (13)	52 (11)	52 (12)	54 (12)	52 (12)	48 (13)
Dropout week 12, No. (%)	57 (7)	13 (7)	5 (5)	11 (13)	12 (8)	16 (7)
Dropout week 24, No. (%)	261 (33)	155 (78)	5 (5)	11 (13)	62 (40)	28 (11)
Pain at baseline, mean (SD)^a^	59 (22)	64 (20)	61 (22)	37 (19)	58 (19)	64 (23)
CRP level at baseline, median (IQR), mg/dL	1.90 (0.82-3.63)	2.19 (0.80-4.20)	1.89 (0.80-3.22)	1.20 (0.80-1.90)	1.25 (0.55-3.22)	2.47 (1.45-4.02)
ESR at baseline, median (IQR), mm/h	43 (32-65)	45 (34-64)	38 (32-53)	30 (28-36)	40 (26-55)	60 (40-85)
**Publicly available data**						
Region	NA	International	International	Central Europe	International	International
Duration, wk	NA	52	24	24	24	52
Treatment vehicle	NA	Injection	Injection	Injection	Injection	Injection
Randomization ratio^b^	NA	4:1	4:1	1:1	2:1	2:1
Start date, y	NA	2005	2005	2008	2005	2006
Washout period, d	NA	28	28	28	14	14
Inclusion/exclusion criteria	NA	Methotrexate, stable for at least 6 mo	Methotrexate, stable for at least 6 mo	Methotrexate, stable for at least 6 mo	Methotrexate, stable	No previous methotrexate treatment allowed

Abbreviations: CRP, C-reactive protein; ESR, erythrocyte sedimentation rate; IQR, interquartile range; NA, not applicable.

SI conversion factor: To convert CRP to milligrams per liter, multiply by 10.

^a^ Pain was measured on a visual analog scale of 1 to 100 mm.

^b^ Randomization ratio expressed as n:1, with n indicating placebo group.

Trial level data included study design and setting, intervention duration, outcomes at week 12 and week 24, general location of the trial (US, European Union, or both), number of treatment arms in the study, original overall trial size, concomitant use of methotrexate or other disease-modifying antirheumatic drugs (DMARDs), approximate years the trial was conducted, and trial design elements (ie, placebo run-in or crossover to active treatment). Patient-level demographic data were basic characteristics of participants (age and sex). The subjective outcome measure was a standard pain severity assessment (0- to 100-mm visual analog scale). Objective measures were levels of the inflammation biomarkers CRP and ESR. For all 3 outcome measures, a negative change from baseline indicated clinical improvement.

In some trials patients initially randomized to placebo treatment were allowed to cross over to the active treatment after week 12. Thus, we a priori selected 12 weeks as a point that was available for most participants preceding the decision point to drop out or change treatment arms. In addition, we compared the outcome at week 12 with the outcome at week 24. Patients who did not continue treatment were excluded from the main analyses because differential effects between weeks 12 and 24 were part of the research question; however, to address the robustness of the findings, the results were compared in a last-observation-carried-forward analysis.

### Statistical Analysis

Log-normally distributed data were log transformed, and statistical tests in these cases were performed in log space. Comparisons between baseline, week 12, and week 24 were performed using paired *t* tests, either in normal space or in log-normal space, if warranted by the data. Baseline data are presented as means (SDs) or, in case of log-normal distribution, as median and interquartile range. Changes from baseline are presented as arithmetic or geometric mean, along with 95% CIs. To investigate whether effects on outcomes were associated, Pearson correlation analyses were performed to test for associations between placebo response (1) at week 12 and week 24, (2) across outcomes at week 12, and (3) between baseline and mean of baseline and week 12 and placebo response at week 12 to analyze influence of baseline on outcomes.^14^ No *P* values were calculated for correlations.

The association between the placebo response and demographic and study variables was investigated using mixed-effects models, with change *Y_ij_* in pain (outcome − baseline), CRP level, or ESR (ratio of outcome to baseline) at week 12 or 24 as the dependent variables, trial as random effect *α_i_*, and baseline, age, sex, washout length, randomization ratio, and use of DMARDs as fixed effects *β_1-6_*. For the *j*th patient in the *i*th trial, the model was

Y*_ij_* = α_0_ = α*_i_* = β_1_baseline_ij_ + β_2_age_ij_ + β_3_sex_ij_ + β_4_washout_i_ + β_5_Rand ratio_i_ + β_6_DMARD_i_ + ε_ij_.

The estimated adjusted effects of the variables were calculated with their 95% CIs, and findings were considered significant if the 95% CI did not cross zero.

All analyses were performed in R, version x64 3.4.1 (R Project for Statistical Computing), using basic functions and the package lm4 for mixed-effects models. Given the low number of hypothesis tests performed in this analysis, we did not correct for multiple testing. *P* < .05 was considered significant.

## Results

**Figure 1.**
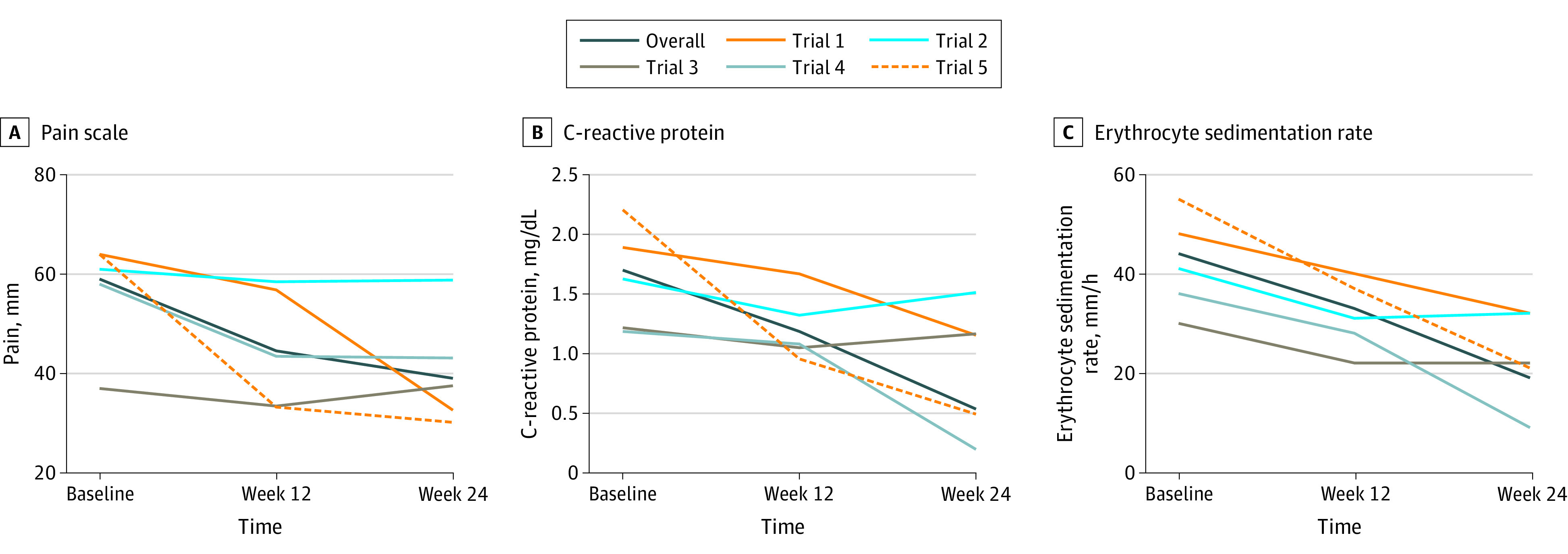
Improvement in Pain Severity, C-Reactive Protein Level, and Erythrocyte Sedimentation Rate in Placebo Arm of 5 Randomized Clinical Trials Values are means for pain (measured by a visual analog scale with values 0-100 mm) (A) and geometric means for C-reactive protein level (B) and erythrocyte sedimentation rate (C) to account for log-normal distribution. All mean changes were significant (*P* < .001).

**Figure 2.**
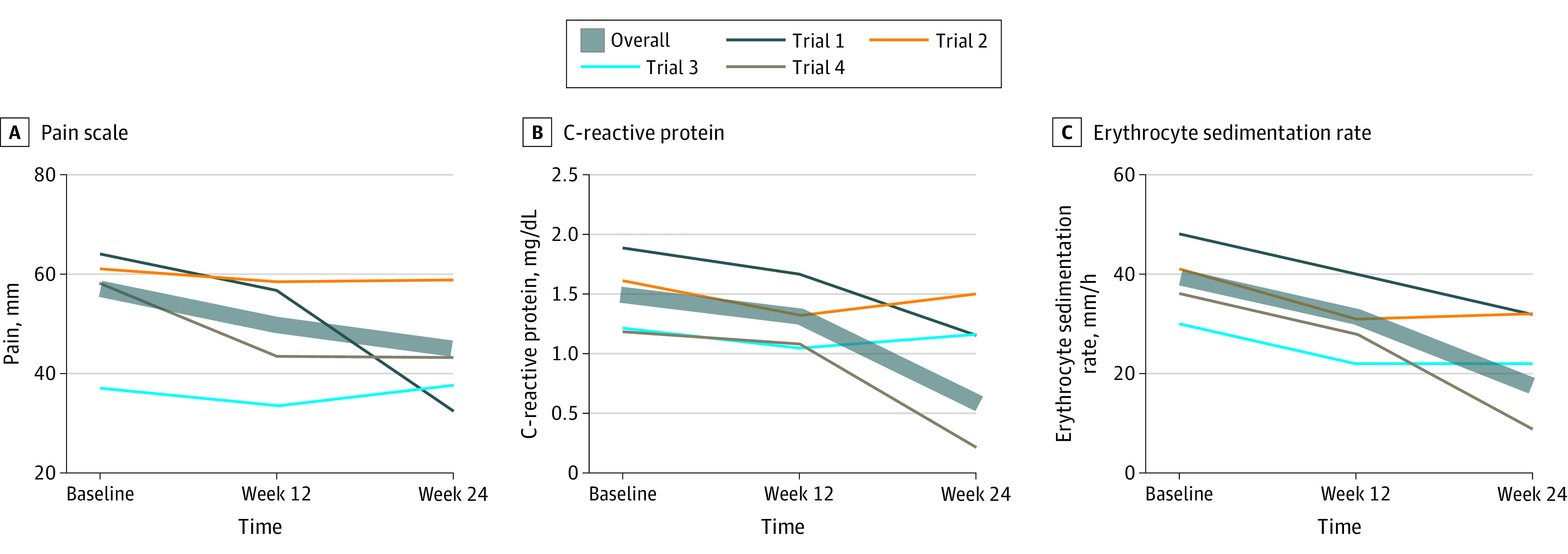
Improvement in Pain Severity, C-Reactive Protein Level, and Erythrocyte Sedimentation Rate for Placebo Arms of Trials 1 Through 4 Values are means for pain (measured by a visual analog scale with values 0-100 mm) (A) and geometric means for C-reactive protein level (B) and erythrocyte sedimentation rate (C) to account for log-normal distribution. All mean changes were significant (*P* < .001).

The data from 5 randomized clinical trials^9,10,11,12,13^ representing 788 participants randomized to placebo control arms were included in this study (644 women [82%]; 144 men [18%]; mean [SD] age, 51 [13] years) (Table 1). All investigations were parallel, double-blind trials of at least 24 weeks’ duration with double-blind placebo administration by vehicle-matched injection. There were statistically significant decreases from high baseline levels in pain intensity at week 12 (−14 mm; 95% CI, −12 to −16 mm) and week 24 (−20 mm; 95% CI, −16 to −22 mm), CRP level at week 12 (−0.51 mg/dL; 95% CI, −0.47 to −0.56 mg/dL) and week 24 (−1.16 mg/dL; 95% CI, −1.03 to −1.30 mg/dL) (to convert to milligrams per liter, multiply by 10), and ESR at week 12 (−11 mm/h; 95% CI, −10 to −12 mm/h) and week 24 (−25 mm/h; 95% CI, −12 to −26 mm/h) (all *P* < .001) (Figure 1). When trial 5, which had the greatest placebo response and included patients naive to DMARDs, was excluded from the analysis (Figure 2), effects were smaller yet remained statistically significant. Pain levels decreased by 7 mm (95% CI, 5-9 mm) at week 12 and 12 mm (95% CI, 10-14 mm) at week 24, CRP levels decreased by 0.18 mg/dL (95% CI, 0.17-0.19 mg/dL) at week 12 and 0.91 mg/dL (95% CI, 0.84-1.00 mg/dL) at week 24, and ESR decreased by 8 mm/h (95% CI, 7-8 mm/h) at week 12 and 22 mm/h (95% CI, 21-23 mm/h) at week 24 (all *P* < .001). Results did not change substantially when last observation carried forward was used instead of exclusion of dropouts, and all results remained significant at *P* < .001.

Correlations between week 12 and week 24 outcomes were moderate to high (pain: *r* = 0.73, CRP: *r* = 0.39, ESR: *r* = 0.59), and those between subjective and objective improvement were lower than those between objective measures (pain/CRP: *r* = 0.27, pain/ESR: *r* = 0.25, CRP/ESR: *r* = 0.48). While correlations between change and baseline were moderate (pain: *r* = 0.47, CRP: *r* = 0.39, ESR: *r* = 0.25), correlations between change and average of baseline and week 12 assessment were poor to nonexistent (pain: *r* = 0.16, CRP: *r* = 0.08, ESR: *r* = 0.02).

In the mixed-effects models, previous treatment with DMARDs (naive vs stable) was identical for all but 1 trial (trial 5) and could therefore not be disentangled from random study effects (Table 2). For all outcomes, baseline values showed a significant influence on the outcome at both weeks 12 and 24. No other variables tested had statistically significant effects, except for washout length, which had an unexpectedly high effect on both CRP level and ESR at week 24 only. This result could be biased: washout length was associated in this data set with earlier use of DMARDs, the only study with no previous use of DMARDs making up 61% of the group of patients with a shorter washout length.

**Table 2.** Effects on Subjective and Objective Outcomes at Week 12 and 24 of Various Factors^a^

Variable	Pain level^b^		C-Reactive protein level^c^		Erythrocyte sedimentation rate^c^	
	Week 12	Week 24	Week 12	Week 24	Week 12	Week 24
Baseline	0.6 (0.5-0.7)^d^	0.6 (0.5-0.7)^d^	−0.001 (−0.002 to −0.001)^d^	−0.001 (−0.002 to −0.001)^d^	−0.006 (−0.007 to −0.004)^d^	−0.005 (−0.007 to −0.002)^d^
Sex	0.7 (−30.5 to 50.0)	0.5 (−40.6 to 50.5)	0.101 (−0.137 to 0.340)	−0.066 (−0.432 to 0.301)	−0.081 (−0.189 to 0.026)	−0.050 (−0.183 to 0.084)
Age	0.1 (−0.1 to 0.3)	0.1 (−0.1 to 0.3)	−0.004 (−0.014 to 0.005)	−0.007 (−0.020 to 0.006)	−0.003 (−0.008 to 0.001)	−0.002 (−0.007 to 0.003)
Randomization ratio	−4.3 (−10 to 10.5)	0.5 (−120.1 to 130.1)	0.004 (−0.164 to 0.172)	0.108 (−0.076 to 0.292)	0.042 (−0.007 to 0.090)	0.025 (−0.038 to 0.089)
Washout length	−0.7 (−10.7 to 0.3)	−0.7 (−20.9 to 10.5)	0.014 (−0.014 to 0.041)	0.036 (0.009 to 0.062)^d^	−0.002 (−0.010 to 0.005)	0.011 (0.001 to 0.021)^d^

^a^ Values are presented as effect on the 0- to 100-mm pain scale or on the ratio outcome to baseline for C-reactive protein level and erythrocyte sedimentation rate to account for log-normal distribution.

^b^ Positive factors indicate improvement.

^c^ Owing to ratio rather than difference calculation, negative factors indicate improvement.

^d^ Statistically significant finding.

## Discussion

Contrary to our hypothesis, we found that objective and subjective outcome measures in rheumatoid arthritis trials improved to a clinically meaningful extent within the 5 trials in this analysis, showing that the placebo responses observed in this study are more than a psychological placebo effect. Correlation analyses of baseline level to placebo response showed moderate correlations; however, correlation between mean of baseline and week 12 and placebo response was poor or nonexistent. This low level could be caused by inclusion biased toward high baseline values, for example, minimum disease severity.^14^ A recent systematic review reinforces this view, finding increased placebo responses in clinical trials of rheumatoid arthritis over the past 30 years and partly attributing these to inflated baseline measures.^20^ On the other hand, a meta-analysis suggested that the method of placebo delivery (eg, oral vs intra-articular) has a significant effect on placebo response in osteoarthritis trials,^21^ showing that the psychological dimension of the placebo response cannot be dismissed. Minimum levels at enrollment for these trials were between 0.63 and 1.47 mg/dL for CRP level or between 28 and 30 mm/h for ESR,^9,10,11,12,13^ which might be the main factors affecting these findings. A minimum pain level was not set as an inclusion criterion for any of the trials; however, the mean baseline level of 59 mm can be considered high and is linked to inflammation severity. Patients were stable with DMARD therapy across trials 1 to 4, while potentially naive patients were introduced to DMARDs in trial 5. Thus, we expected a higher response in the placebo arms of these trials due to the drug effect of DMARDs. We performed a separate analysis excluding this trial, yet the results stayed significant. While the magnitude of placebo response was clinically meaningful across pain, CRP level, and ESR, these findings are not necessarily in the same patients: correlations between improvement of pain and CRP level and ESR were only approximately 0.25. Even for the 2 objective inflammation markers (ESR and CRP level), the correlation was below 0.5. The correlation between week 12 and week 24 was moderate to high, showing that natural history, standard of care, and within-patient variability might explain another important part of the placebo response.

The clinical phase of rheumatoid arthritis in its natural history progresses slowly,^15^ making it difficult to compare with progression within a clinical study. However, CRP levels seem to increase even before clinical manifestation,^16,17^ and improvement of the degree found in this study seems unlikely to happen as spontaneous, untreated remission.^18^

### Limitations

Given that this was a retrospective study based on available deidentified data, there are several limitations, such as demographic characteristic variables. Only a comparison of placebo arms with natural history arms could have clearly demonstrated the influence of regression to the mean, and such arms were missing in the trials included in our analysis. A no-treatment group would have yielded important information on the natural history of outcome fluctuation in association with placebo response. Most patients were randomized to placebo plus standard of care, that is, DMARDs such as methotrexate, which represents a typical situation in rheumatoid arthritis trials. Thus, we cannot rule out DMARDs increasing the placebo response; however, the placebo response noted with pain severity was similar to other causes of chronic pain,^19^ which mirrors the typical situation in rheumatoid arthritis trials.

## Conclusions

The results of this study suggest that, to reduce the placebo response in clinical trials, replacing subjective with objective outcomes will not necessarily lead to clearer results. Within our data set, high baseline values at enrollment due to minimum levels as inclusion criteria and natural history seem to overlay the psychological placebo effects. Appropriate means to account for nonpsychological elements of the placebo response could be using alternative study designs, for example, with multiple baseline measures,^22^ or using methods to statistically account for the regression to the mean in the analysis.^23^ Including no treatment arms in the RCTs to understand the role of natural history and regression to the mean more clearly would be beneficial for future trials, and similar comparisons between objective and subjective outcomes in other diseases may corroborate or challenge our findings. For new drug development, efforts to understand how baseline covariates and confounding factors in the placebo responses via data sharing initiatives^24,25^ can be helpful to study design considerations, expedite clinical drug development, and ultimately bring effective treatments to patients in need.
